# LRRK2 at the Interface Between Peripheral and Central Immune Function in Parkinson’s

**DOI:** 10.3389/fnins.2020.00443

**Published:** 2020-05-21

**Authors:** Rebecca L. Wallings, Mary K. Herrick, Malú Gámez Tansey

**Affiliations:** ^1^Department of Neuroscience and Center for Translational Research in Neurodegenerative Disease, University of Florida College of Medicine, Gainesville, FL, United States; ^2^Laney Graduate School, Emory University, Atlanta, GA, United States

**Keywords:** Parkinson’s disease, LRRK2, immune function, inflammation, systemic inflammation

## Abstract

It is becoming increasingly accepted that there is an interplay between the peripheral immune response and neuroinflammation in the pathophysiology of Parkinson’s disease (PD). Mutations in the *leucine-rich-repeat kinase 2* (*LRRK2*) gene are associated with familial and sporadic cases of PD but are also found in immune-related disorders, such as inflammatory bowel disease (IBD) and leprosy. Furthermore, LRRK2 has been associated with bacterial infections such as *Mycobacterium tuberculosis* and *Salmonella typhimurium*. Recent evidence suggests a role of LRRK2 in the regulation of the immune system and modulation of inflammatory responses, at a systemic level, with LRRK2 functionally implicated in both the immune system of the central nervous system (CNS) and the periphery. It has therefore been suggested that peripheral immune signaling may play an important role in the regulation of neurodegeneration in LRRK2 as well as non-LRRK2-associated PD. This review will discuss the current evidence for this hypothesis and will provide compelling rationale for placing LRRK2 at the interface between peripheral immune responses and neuroinflammation.

## Introduction

Parkinson’s disease (PD) is a complex, multifactorial neurodegenerative disease. The aetiology of PD is largely unknown, thought to involve a complex interaction between various genetic and environmental factors. Although typically thought of as a disease limited to the central nervous system (CNS), evidence has accumulated in recent years suggesting a crucial and fundamental role of neuroinflammation in the pathogenesis of PD. Although mainly associated with the presence of activated microglia and elevated cytokine levels in the CNS, active participation of the peripheral immune system has also been noted with infiltration and reactivation of peripheral immune cells into the CNS as a potential mechanism that could exacerbate neuroinflammation and perpetuate the neurodegenerative process.

Mutations in the *LRRK2* gene are the most frequent cause of familial PD (Singleton et al., 2013), with seven pathogenic mutations, which cluster around the catalytic domains of the protein, currently identified. Clinically, mutant *LRRK2*-PD patients are often considered indistinguishable from sporadic and idiopathic patients (Haugarvoll and Wszolek, 2009; Gatto et al., 2013). Therefore, deciphering the role of LRRK2 in PD pathogenesis may reveal common pathological mechanisms underlying idiopathic PD and is consequently of great research importance.

LRRK2 is expressed in both innate and adaptive immune cells and this expression is tightly regulated by immune stimulation. LRRK2 is a member of the receptor interacting protein (RIP) kinase family, which are a group of proteins that detect and respond to cellular stress by regulating cell death and activation of the immune system (Rideout and Re, 2017), highlighting a potential role of LRRK2 in immune system regulation. This is supported by reports biochemically linking LRRK2 to the pathways regulating inflammation, autophagy and phagocytosis in immune cells (Wallings and Tansey, 2019). Furthermore, polymorphisms in the LRRK2 gene have been linked to inflammatory diseases such as leprosy and the IBD, Crohn’s disease (CD), highlighting a critical role of LRRK2 in inflammation.

This review will outline the current evidence for the presence of systemic inflammation in PD, and what is currently understood about the role of LRRK2 in both central and peripheral immune cells. Furthermore, we discuss evidence that implicates LRRK2 as a mediator of the cross-talk between the central and peripheral immune system at both the blood–brain barrier (BBB) and the gut-brain axis. Such evidence makes LRRK2 an appealing target for future therapeutics aimed at curbing inflammation. However careful consideration must be taken when targeting LRRK2-kinase activity levels in the periphery, as will be discussed.

### Systemic Inflammation in Parkinson’s Disease

Once thought to be immune-privileged, it is now clear that the brain has its own resident immune cells and that there is extensive bi-directional communication with the peripheral immune system. Some of these bi-directional communications between the CNS and the peripheral immune system have been shown to be critical in maintaining healthy brain function and may be important for learning and memory (Filiano et al., 2015; Louveau et al., 2015; Kipnis, 2016). Activation of immune cells is a healthy response to protect and repair the body; however, chronic activation and therefore chronic inflammation is deleterious and damaging. Brain-resident microglia can become chronically activated with increasing age, traumatic brain injury, and in response to chronic systemic disease. Detrimental neuroinflammation ensues from such chronic activation and is believed to compromise neuronal survival and promote circuit dysfunction.

The first observation supporting a role of neuroinflammation in PD came from *post-mortem* analysis which reported the presence of human leukocyte antigen DR isotype (HLA-DR) positive reactive microglia in the *substantia nigra pars compacta* (SNpc) of PD patients (McGeer et al., 1988). Alterations in cytokine levels have been observed in PD brains, with elevated immunoreactivity of interleukin-1β (IL-1β), interleukin-2 (IL-2), interleukin-6 (IL-6), tumour necrosis factor (TNF), and transforming growth factor-β1 (TGF-β1) detected specifically in the striatal dopaminergic regions of PD brains (Mogi et al., 1994a, b). In the SNpc of PD patients, a significant increase in the density of glial cells expressing TNF, Il-1β, and interferon-γ (IFN-γ) has been reported relative to controls (Hunot et al., 1999). In agreement with these findings, elevated levels of TGF-β1, IL-6, and IL-1β have been observed in the cerebrospinal fluid (CSF) of PD patients (Chen et al., 2018). Furthermore, inflammatory biomarkers correlate with more severe motor symptoms and cognitive impairment in PD, indicating an association between inflammation and more aggressive disease course (Hall et al., 2018). Such findings suggest increased neuroinflammation in PD brains.

For the last two decades research has focused on neuroinflammation processes involved in PD. However, it is becoming increasingly evident that peripheral inflammatory responses contribute to PD pathogenesis (Gelders et al., 2018; Skaper et al., 2018). For example, reports have demonstrated that levels of inflammatory cytokines, such as TNF (Bu et al., 2015; Williams-Gray et al., 2016), IL-1β (Bu et al., 2015; Dursun et al., 2015; Hu et al., 2015) and IL-6 (Bu et al., 2015; Dursun et al., 2015; Williams-Gray et al., 2016), are elevated in the serum of PD patients (Qin et al., 2016). Alterations in cytokine receptors have also been noted, with serum levels of TNF and the soluble forms of their receptors (sTNFRs) significantly increased in patients with PD relative to healthy controls (McCoy et al., 2006) which was associated with a later disease onset (Scalzo et al., 2010). In addition, alterations in immune cell subsets in peripheral blood of PD patients have been reported. For example, increased classical monocytes have been observed in peripheral blood of PD patients (Grozdanov et al., 2014). As well, monocytes from PD patients exhibit an increased response to the toll-like receptor 4 (TLR4) ligand, lipopolysaccharide (LPS) and display a distinct transcriptome signature and inflammatory profile relative to healthy controls (Grozdanov et al., 2014). In conjunction with this, increased number of pro-inflammatory Th17 cells have been found in peripheral blood from newly diagnosed PD patients (Chen X. et al., 2017; Yang et al., 2017). Similarly, PD patients have been reported to show a predominant expression of CD8^+^ T cells and an increase in the ratios of IFN-γ-producing to IL-4-producing T cells (Baba et al., 2005). Increased effector/memory T cells have also been reported, with this elevation correlating with scores on the Unified Parkinson’s Disease Rating Scale III (UPDRS-III) (Saunders et al., 2012). Similarly, D1-like and D2-like dopamine receptor expression on CD4^+^ naïve T cells is also correlated with scores on the UPDRS-III (Kustrimovic et al., 2016). Interestingly, α-synuclein peptides can trigger helper and cytotoxic T cells to secrete cytokines, including IFN-γ, IL-2, and IL-5 (Sulzer et al., 2017). In addition, one of these peptide regions strongly binds to major histocompatibility complexes encoded by HLA (DRB1^∗^15:01, DRB5^∗^01:01) that are associated with PD by genome-wide association studies (GWAS) (Hamza et al., 2010; Greenbaum et al., 2011; Wissemann et al., 2013; Hill-Burns et al., 2014; Kannarkat et al., 2015). Collectively this data supports the idea that systemic inflammation is important to, and may contribute to, the pathogenesis of PD.

Circulating peripheral monocytes are known to enter tissue, including the brain, during active disease states and mediate pro and anti-inflammatory responses. A key regulatory mechanism for tissue entry is the monocyte chemoattractant protein, CCL2. Interestingly CCL2 has been observed to be elevated in both the blood and CSF of PD patients (Reale et al., 2009; Grozdanov et al., 2014), suggesting increased infiltration of peripheral monocytes in the brains of PD patients. Evidence from animal models of PD support a role of peripheral immune cell CNS-infiltration in pathogenesis. For example, it has been demonstrated in a viral mouse model overexpressing human α-synuclein that dopaminergic neuronal loss is dependent on peripheral monocyte infiltration into the CNS. Genetic deletion of the chemokine receptor that interacts with CCL2, CCR2, prevents monocyte entry and blocks neuronal degeneration (Harms et al., 2018). Furthermore, it has also been reported that α-synuclein fibrils, but not the monomeric species, are able to recruit peripheral monocytes and macrophages into the brain, causing increased microglia activation and axonal loss in the striatum of wild-type (WT) rats (Harms et al., 2017). However this has not been replicated in an acute MPTP (1-methyl-4-phenyl-1,2,3,6-tetrahydropyridine) model of PD, which demonstrated that CCR2^+^ monocytes did not contribute to dopaminergic neuronal loss (Parillaud et al., 2017). Whilst the MPTP-toxin model of PD is a useful for tool for the rapid study of the consequences and mechanisms of dopamine dysfunction *in vivo*, it is unable to capture the insidious and progressive effects of PD. Given that peripheral immune cell infiltration into the CNS is likely to be an early event in disease (Johnson et al., 2019), this may account for the inability of this model to replicate such results. Collectively, these studies provide evidence that the inflammation in the CNS involves both microglia and peripheral immune cells prior to neurodegeneration, and peripheral immune cell infiltration may be instrumental in PD progression.

GWAS provide additional support for the importance of an immunological mechanism driving disease, showing that polymorphisms in the HLA-DR locus, which encodes for the major histocompatibility complex class II (MHC-II) that is involved in antigen presentation, are associated with sporadic, late-onset PD (Hamza et al., 2010). Furthermore, GWAS has more recently identified 17 novel loci which overlap between PD and autoimmune diseases, including known PD loci adjacent to *GAK*, *HLA-DRB5*, *LRRK2*, and *MAPT* for rheumatoid arthritis and IBD (Witoelar et al., 2017). Epidemiological studies have suggested that the incidence of PD development is decreased in long-term users of non-steroidal anti-inflammatory drugs (Chen et al., 2003; Wahner et al., 2007). In addition, ibuprofen has been highlighted in a meta-analysis to provide significant protection from PD (Gao et al., 2011). This data from genetic and epidemiological studies, coupled with the *post-mortem* and biochemical data previously discussed, provide compelling evidence for a fundamental role of systemic inflammation in PD.

### LRRK2 Expression in Cells of the CNS and Peripheral Immune Cells

As PD has typically been thought of as a disease limited to the CNS, research has overwhelmingly focused on the role of LRRK2 and the effects of *LRRK2* mutations in neurons. However, LRRK2 expression is considerably lower in the brain relative to organs in the periphery (Biskup et al., 2007; Melrose et al., 2007; Westerlund et al., 2008), with low *LRRK2* gene expression observed in both human neurons and astrocytes (Zhang et al., 2016). Furthermore, although detected, LRRK2 immunoreactivity is reportedly weak in neurons of the SNpc and cortex of *post-mortem* PD brains (Dzamko et al., 2017).

Astrocytes provide an important contribution during neuroinflammatory responses. These cells are able to become reactive and work as immune mediators in the brain when elicited by proper stimuli. Thus far, inconsistent reports have been published regarding the expression of LRRK2 in astrocytes. For example, although *post-mortem* PD brain analysis suggests that LRRK2 is expressed in astrocytes (Dzamko et al., 2017), *LRRK2* mRNA could not be unequivocally identified in astrocytes in *post-mortem* brains of healthy controls and protein expression was only noted in occasional glial cells with astrocytic morphology (Sharma et al., 2011). Whether LRRK2 expression increases in astrocytes of PD-brains relative to healthy controls remains to be empirically determined. Despite low expression levels, it is increasingly evident that LRRK2 may play a functional role in astrocytes. For example, TGFβ1, which has been shown to inhibit microglial inflammatory responses in a rat model of PD (Chen S. et al., 2017), and matrix metallopeptidase 2 (MMP2), which has been shown to degrade α-synuclein aggregates (Oh et al., 2017), were found to be down-regulated in LRRK2-*G2019S* astrocytes derived from patient-induced pluripotent stem cells (iPSC) (Booth et al., 2019). Furthermore, overexpressing *G2019S*, *R1441C* or *Y1699C-LRRK2* impairs the lysosomal degradation capacity of primary mouse astrocytes (Henry et al., 2015) which may promote α-synuclein accumulation and propagation. Further research is still required in order to investigate the expression levels and role of LRRK2 in astrocytes in order to understand how potential non-cell-autonomous processes contribute to development of disease pathology [reviewed in detail in Booth et al. (2017)].

Under homeostatic conditions LRRK2 expression is also low or absent in microglia, as seen *post-mortem* in healthy control brains (Miklossy et al., 2006; Sharma et al., 2011). Although increased LRRK2 levels have previously been observed in response to LPS in primary murine microglia (Moehle et al., 2012), it has been demonstrated that, despite inducing neuronal loss in the SNpc, *in vivo* LPS treatment failed to increase microglia LRRK2 protein levels in *R1441C* and *G2019S* mice (Kozina et al., 2018). Similar results have been observed *ex vivo*, with neither LPS (Russo et al., 2015) or priming with α-synculein pre-formed fibrils (PFFs) (Russo et al., 2019) increasing LRRK2 protein expression in cultured murine microglia. Collectively, these results suggest that LRRK2 levels in microglia may not have a direct effect on neuroinflammation in PD. It has been suggested that impaired peripheral immune cell functions, as a consequence of *LRRK2* mutations, have a deleterious impact on brain microglia and dopaminergic neurons as a secondary effect. The role of peripheral immune cell activation and cytokine release on the CNS in LRRK2 models will be discussed later in this review.

It is notable that a loss of *Lrrk2* is insufficient to induce neurodegeneration in rodent models of disease (Hinkle et al., 2012; Tong et al., 2012). Similarly, no changes in LRRK2 expression levels were observed in the brains of PD patients (Dzamko et al., 2017), despite changes being reported in the periphery in other studies, as discussed below. Collectively such findings suggest that LRRK2 may exert its effects on PD in areas outside of the CNS. LRRK2 expression has been observed in peripheral immune cells, with LRRK2 expression increasing in response to pro-inflammatory signals, strongly implicating LRRK2 as a regulator of these immune responses. For example, increased LRRK2 expression in response to microbial pathogens has been observed in human B cells, T cells, macrophages and non-classical monocytes (Gardet et al., 2010; Hakimi et al., 2011; Thévenet et al., 2011; Moehle et al., 2012; Kuss et al., 2014; Cook et al., 2017). Furthermore, LRRK2 is upregulated in unstimulated sporadic-PD neutrophils (Atashrazm et al., 2019), B cells, T cells and non-classical monocytes which is accompanied by increased secretion of pro-inflammatory cytokines from monocytes and T cells (Cook et al., 2017) [reviewed in detail in Lee et al. (2017) and Wallings and Tansey (2019)].

In addition to LRRK2 expression levels, recent investigations into the role of LRRK2 kinase activity in peripheral immune cells and microglia have been reported. For example, it has been reported that increased phosphorylated LRRK2 at s935, an indirect autophosphorylation site of LRRK2, is observed in human peripheral blood monoculear cells (PBMCs) upon stimulation with an immune stimulation cocktail of phorbol 12-myristate 13-acetate (PMA) and IFN-γ (Thirstrup et al., 2017). Similarly, toll-like receptor 2 (TLR2) and TLR4 stimulation have been shown to increase LRRK2 phosphorylation on s935 in bone-marrow derived macrophages (BMDMs) from WT mice (Dzamko et al., 2012). However, it was also observed that *Lrrk2* knock-out (KO) macrophages do not have an altered pattern of pro-inflammatory cytokines secretion after TLR2 or TLR4 stimulation, indicating that LRRK2 function might be regulated by PAMP signaling without affecting downstream cytokine responses.

With regards to cells of the CNS it has recently been shown that nigrostriatal dopamine neurons from healthy controls express extremely low basal levels of LRRK2 phosphorylated at s1292. However, detectable levels of pS1292 signal was observed in nigral microglia (Di Maio et al., 2018). It seems therefore that LRRK2-kinase activity may be increased in microglia relative to neurons. Interestingly however, surviving dopamine neurons and also microglia from idiopathic-PD patients had a significant increase in phosphorylated LRRK2 at s1292 and phosphorylation of the LRRK2 substrate Rab10 at t73 relative to healthy controls. It seems therefore that, although exhibiting low activity levels in healthy neurons, that endogenous WT LRRK2 is activated in dopamine neurons in idiopathic PD.

Increased kinase activity associated with *LRRK2* mutants has been linked to pathological function of LRRK2 in disease. Peripheral pro-inflammatory cytokine levels are higher in a percentage of asymptomatic subjects carrying the *G2019S-LRRK2* mutation (Dzamko et al., 2016), which consistently increases LRRK2 kinase activity (Smith et al., 2006; West et al., 2006; Luzon-Toro et al., 2007; Anand and Braithwaite, 2009; Covy and Giasson, 2009), suggesting an early role of inflammation in the periphery in disease. Interestingly, increased phosphorylated s1292 proximity ligation signal, indicative of increased LRRK2 kinase activity, has been reported in the nigral microglia of idiopathic PD cases as well rodents treated with rotenone, a pesticide used to model PD due to its selective degeneration of the nigrostriatal pathways (Di Maio et al., 2018). Similarly, increased expression of phosphorylated LRRK2 on s935 has been observed in the PBMCs of idiopathic-PD patients (Dzamko et al., 2013). Surprisingly it has recently been reported that the PBMCs of *G2019S*-carriers with manifesting PD exhibit a decrease in LRRK2 phosphorylated on s935 relative to non-manifesting *G2019S*-carriers and idiopathic patients (Perera et al., 2016). The s935 residue of LRRK2 is proposed to be a constitutive phosphorylation site that is amenable to regulation by LRRK2 kinase activity through other kinases and signaling pathways (Zhao et al., 2012). Given the higher kinase activity of G2019S-LRRK2, the decrease in s935 expression in *G2019S* patients may therefore reflect compensatory biological mechanism that lead to de-phosphorylation of the s935 residue in disease manifesting carriers. Similarly, reduced LRRK2 s910 and s935 phosphorylation has also been observed in *post-mortem* brain tissue from patients with idiopathic PD (Dzamko et al., 2017), suggesting a potential pathogenic role for these residues in PD. A significant age-dependent reduction in astrocytes in the striatum of LRRK2 s910/s935 phosphorylation deficient mice inoculated with α-synuclein PFFs with concomitant increased α-synuclein accumulation has previously been reported (Zhao et al., 2018). It was suggested that the reduction in astrocytes may promote α-synuclein accumulation and propagation.

It has recently been suggested that LRRK2 may promote mitochondrial fission via Drp1 in a kinase-dependent manner, with increased fission due to *G2019S-LRRK2* expression resulting in increased TNF-α production in the brains of mice (Ho et al., 2018). Brain lysates of *G2019S-LRRK2* knock-in mice exhibited reduced NFkB p50 s337 phosphorylation, decreasing NFkB p50 inhibitory signaling and pro-inflammatory gene transcription, compared to WT mice (Russo et al., 2018). Such results strongly implicate a role of increased LRRK2 kinase activity levels in microglia and peripheral immune cells in a disease relevant manner [the role of LRRK2 kinase activity in various signaling pathways in different immune cell subsets has been reviewed recently in detail in Wallings and Tansey (2019)].

Recently, transcriptome analysis of *Lrrk2*-KO microglia cells revealed altered inflammatory related pathways upon α-synuclein fibril treatment (Russo et al., 2019). This data suggested that, whilst *Lrrk2*-KO microglia had only a subtle influence on basal gene expression, this effect became more pronounced upon treatment with α-synuclein preformed fibrils (PFFs) or LPS. Furthermore, phosphorylated s935 LRRK2, an indirect readout of LRRK2 kinase activity, was increased upon inflammatory insults in primary microglia from WT mice, suggesting LRRK2 is influenced by intracellular signaling of microglia after a PD-related insult. Such data is also in keeping with the “multiple-hit” hypothesis which suggests that PD is triggered by environmental factors, such as bacterial and viral infection and microbiome perturbation, and is subsequently facilitated and exacerbated by factors such as genetics and aging (Johnson et al., 2019; Patrick et al., 2019). Collectively, such data highlights LRRK2 and its kinase activity as a mediator of inflammatory responses in both the periphery and the CNS. It is therefore possible that LRRK2 is a potential regulator of the crosstalk between periphery and the CNS, and may lie center stage of the inflammation observed in PD.

### LRRK2 Expression in Peripheral Organs

It is important to note that LRRK2 is expressed highly in peripheral organs such as the lung, spleen and kidneys, relative to the brain (Biskup et al., 2007; Melrose et al., 2007; Westerlund et al., 2008). With regards to the kidneys, although not typically implicated in PD pathology, proper kidney function is implicated in immune function, with the removal of cytokines from the blood limiting inflammation (Betjes et al., 2013; Kurts et al., 2013), and the clearance of bacterial components reducing would−be immune cell activation by pattern recognition receptors (PRRs) (Betjes, 2013; Hato and Dagher, 2015). *Lrrk2*-KO rats exhibit enlarged kidneys with pigment accumulation and irregular hyaline droplets, indicative of irregular phagocytic activity, in proximal tubule endothelial cells (Baptista et al., 2013). *Lrrk2*-KO animals have been reported to show dramatic α-synuclein pathology in the kidneys, as well as biphasic, age-dependent changes in autophagy proteins (Tong et al., 2010). Collectively, such data highlights an important role of LRRK2 in proper kidney function.

Regarding LRRK2 in the lungs, studies in *Lrrk2*-KO mice have found morphological and histopathological abnormalities in lung tissue that have been associated with impairments in the autophagy pathway (Tong et al., 2010, 2012; Hinkle et al., 2012). Increased number and size of lamellar bodies has also been found in the lungs of *Lrrk2*-KO but not kinase-dead (KD) mice, suggesting that the LRRK2 protein-protein binding domains, rather than the kinase domain, may be crucial for normal lung function (Tong et al., 2012). However, inhibition of the LRRK2 kinase domain in non-human primates induces abnormal cytoplasmic accumulation of secretory lysosome-related organelles known as lamellar bodies in type II pneumocytes of the lung (Fuji et al., 2015), which has also been observed in the lungs of 16-month old *Lrrk2*-KO rats (Baptista et al., 2013). Lamellar bodies are the secretory organelles that store surfactant, which play a pivotal role in innate immunity of the lung (Takahashi et al., 2006). Given that LRRK2 is also associated with infections of the lung such as tuberculosis (discussed later in this review), it appears that LRRK2 expression, perhaps specifically LRRK2 kinase activity, may be crucial for immunity in the lung.

The spleen plays multiple supporting roles in the body, such as filtering blood as part of the immune system, storing white blood cells, and helping fight bacteria. Interestingly, *Lrrk2*-KO leads to alterations in the cellular composition of the spleens of rats, with an increase in the number of CD4^+^ helper T cells and CD11b^+^ monocytes and a decrease in B cells (Ness et al., 2013). However, splenocytes from *Lrrk2*-KO mice infected with Rat adapted Influenza Virus (RAIV) and *Streptococcus pneumoniae* exhibit decreased CD11b^+^ monocytes and increased CD8^+^ cytotoxic T cell numbers. Such data suggests that a loss of LRRK2 may alter host resistance to infection.

**FIGURE 1:**
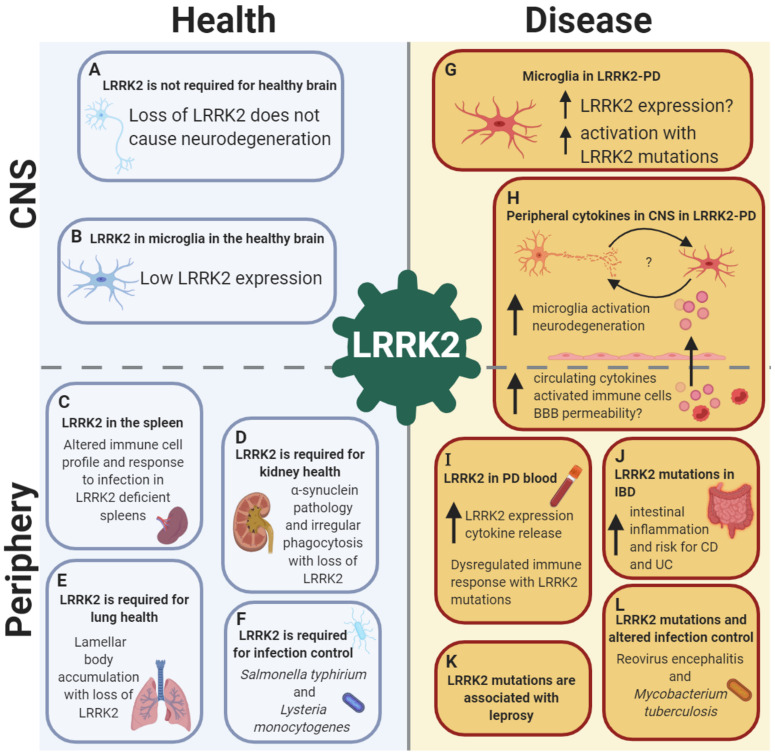
LRRK2 is implicated in health and disease in both the CNS and the periphery, with a crucial role in the immune system. LRRK2 in the healthy brain: **(A)** LRRK2 may not be essential for neuronal development as global LRRK2 deficiency in rodents is not accompanied by neurodegeneration in dopamine-striatal and other pathways. **(B)** In the healthy brain, LRRK2 is absent or expressed at low levels in microglia, suggesting a minimal role of LRRK2 in brain-resident innate immune cells under homeostatic conditions. Role of LRRK2 in health in peripheral organs and the immune system: **(C)** LRRK2 is required for spleen, **(D)** kidney and **(E)** lung health, as well as **(F)** pathogen control and host response to infections such as *Salmonella typhirium* and *Lysteria monocytogenes.* LRRK2 in the brain in PD: **(G)** In LRRK2-PD, LRRK2 expression is increased in microglia, with increased activation of microglia observed with *LRRK2*-PD mutations. **(H)** LRRK2 may exert its effects on the brain from the periphery in PD, with increased circulating cytokines potentially increasing BBB permeability with LRRK2 mutations, causing microglia activation and neurodegeneration, leading to bi-directional interplay between neuronal death and microglia priming. Role of LRRK2 in disease in peripheral organs and the immune system: **(I)** LRRK2 expression is increased in peripheral immune cells in both LRRK2 and non-LRRK2 PD, with concomitant increases in cytokine release. **(J)** LRRK2 is associated with gut inflammation, with an increased risk of both Crohn’s disease (CD) and ulcerative colitis (UC) with mutations. **(K)** LRRK2 risk and protective genetic variants are associated with the infectious and autoimmune disease leprosy. **(L)** LRRK2 mutations have been shown to alter infection control and host response to Reovirus encephalitis and *Mycobacterium tuberculosis.* Created with BioRender.com.

Collectively, these data suggest that LRRK2 has a diverse functional role in many organs outside of the CNS, and is required for the healthy function of organs such as the lung and kidneys, and may be associated with efficient host responses to infections (Figure 1). Such data has great implications on the use of LRRK2 kinase inhibitors for PD treatment, as such inhibitors may have deleterious and harmful effects on health in the periphery.

### LRRK2 Is Associated With Infection and Inflammation

Peripheral infections may enhance neurodegeneration either via direct toxicity of bacterial or viral toxins, or by circulating cytokines. PD patients with viral or bacterial infections exhibit deterioration of both motor and cognitive function, suggesting that inflammation caused by infections may be deleterious and a contributor to disease (Brugger et al., 2015). Interestingly, the highly pathogenic H5N1 avian flu virus is capable of entering the CNS and induces neuroinflammation via microglia activation and increases α-synuclein aggregation in mice (Jang et al., 2009). Furthermore, the H1N1 influenza virus has been observed to have synergistic effects with MPTP, leading to increased SNpc dopaminergic neuronal loss than MPTP treatment alone, which could be eliminated by influenza vaccination or treatment with anti-viral medication (Sadasivan et al., 2017). Such observations further support the hypothesis that CNS disorders of protein aggregation such as PD can be initiated or exacerbated by bacterial and viral pathogens.

A role of LRRK2 in regulating inflammation and pathogen defense has been suggested by reports implicating LRRK2 in several bacterial infections. For example, meta-analysis of human gene expression identified the *LRRK2* pathway to be significantly enriched in response to *Mycobacterium tuberculosis* (Mtb) infection, with *LRRK2* being a highly significant differentially enriched gene (DEG) (Wang et al., 2018). This is supported by the observation that a loss of LRRK2 enhances Mtb control and decreases bacterial burdens in both primary mouse macrophages and human iPSC−derived macrophages (Hartlova et al., 2018). LRRK2 has also been implicated in the control of the enteric pathogen *Salmonella typhimurium* via NLRC4 inflammasome regulation in macrophages from *Lrrk2-*KO mice (Gardet et al., 2010; Liu et al., 2017). Furthermore, paneth cells from *Lrrk2-*KO mice are more susceptible to infection from *Listeria monocytogenes*, with a loss of Lrrk2 decreasing lysozyme levels, an antimicrobial enzyme responsible for the degradation and lysis of bacteria (Zhang Q. et al., 2015). Whilst the precise mechanisms underlying the regulation of pathogens via LRRK2 remains to be determined, it has been suggested that these may be dependent on sex, pathogen type and cell-type (Herbst and Gutierrez, 2019; Shutinoski et al., 2019).

Polymorphisms in the *LRRK2* gene have been linked to increased susceptibility to leprosy (Fava et al., 2016). Of particular interest is the recently reported antagonistic, pleiotropic effects of LRRK2 in PD and leprosy type-1 reactions (T1R), with the gain-of-kinase function *R1628P* mutation found to be a risk-variant for PD but as protective for T1R (Fava et al., 2019). This *R1628P* mutation reduces apoptosis, with apoptotic cells known to increase inflammation locally (Yang et al., 2015) as well as release multiple anti-inflammatory mediators (Zhang L. et al., 2015). It was therefore hypothesized that the resulting reduction in anti-inflammatory molecules in the CNS would be disease promoting, whilst the decrease in apoptotic debris is protective in leprosy patients. Similar antagonistic pleiotropic effects of the gain-of-kinase function *G2019S* mutation have recently been reported in models of *S. typhimurium*-induced sepsis and reovirus-induced encephalitis (Shutinoski et al., 2019). It was observed that the *G2019S* mutation controlled *S. typhimurium* infection better, with reduced bacterial growth and longer survival during sepsis; an effect which was dependent on myeloid cells. However, animals with reovirus-induced encephalitis that expressed the *G2019S* mutation exhibited increased mortality, increased reactive oxygen species and higher concentrations of α-synuclein in the brain. Such data implies potential opposing effects of LRRK2 kinase-mediated inflammation in the CNS versus the periphery.

### LRRK2 and the Gut-Brain Axis

The gut-brain axis describes the bidirectional communication between the central and enteric nervous and endocrine systems, as well as the regulation of immune responses in the gut and the brain (Houser and Tansey, 2017). The microbiome is particularly concentrated in the gastrointestinal tract, and is now known to influence the systems incorporated in the gut-brain axis (Mulak and Bonaz, 2015; Ghaisas et al., 2016; Kowalski and Mulak, 2019). Furthermore, gut microbiota upregulates local and systemic inflammation through different mechanisms including the release of lipopolysaccharides from pathogenic bacteria (Villaran et al., 2010). Gut bacteria are also able to produce numerous neurotransmitters and neuromodulators, such as short chain fatty acids, however the role of these in neuroinflammation is not yet fully understood (Mulak, 2018).

In the last few years, there have been new findings identifying a relationship between gut microbiome dysbiosis and PD (Lin et al., 2018; Sun and Shen, 2018; Mulak et al., 2019). For example, analysis of immune profiles of stool from PD patients revealed increased levels of intestinal inflammation in PD patients, as well as greater incidence of intestinal disease and digestive problems (Houser et al., 2018). Reduced abundance of the bacteria *Prevotellaceae* has been reported in fecal samples from PD patients (Scheperjans et al., 2015). Interestingly, low levels of this bacteria increases gut permeability leading to increased enteric nervous system (ENS) environmental exposure and increased α-synuclein expression in the colon (Bedarf et al., 2017). Such increases in α-synuclein have been proposed to function as a messenger to alert the immune cells in the CNS to the presence of certain pathogens (Fitzgerald et al., 2019). For example, recent studies have reported aggregated α-synuclein causing activation and migration of neutrophils, microglia and dendritic cells in the CNS (Sampson et al., 2016; Sun and Shen, 2018). Furthermore, *Prevotellaceae* is believed to be important for not only maintaining healthy gut but also healthy BBB (Keshavarzian et al., 2015), which may increase circulating cytokine permeability into the CNS, as will be discussed later in this review. Collectively, such data suggests that gut microbiome dysbiosis may be instrumental in systemic inflammation and the aetiology of PD.

CD and ulcerative colitis (UC) are the two major subtypes of IBD and are associated with inflammation in different regions of the gut. Interestingly, patients with IBD have a 22% increased incidence of PD compared to non-IBD individuals (Villumsen et al., 2019). It has recently been demonstrated that early exposure to anti-TNF therapy is associated with substantially reduced PD incidence in individuals with IBD, highlighting systemic inflammation as a potential link between these two diseases (Peter et al., 2018). Interestingly, genetic variances and mutations in the *LRRK2* gene have been demonstrated to increase the incidence of PD in both CD (Witoelar et al., 2017) and UC patients (Villumsen et al., 2019). Furthermore, *LRRK2* has been identified by GWAS as a major susceptibility gene for CD (Liu et al., 2015; Hui et al., 2018). The *LRRK2* risk allele, *N2081D*, is located in the kinase domain and is associated with increased kinase activity, whereas the protective variants, *N551K* nor *R1398H*, had no effects on kinase activity. Interestingly, the protective variant *R1398H* was shown to increase GTPase activity, deactivating LRRK2. Furthermore, the PD-associated *G2019S* mutation has been shown to be increased in CD patients in the Ashkenazi Jewish population (Rivas et al., 2019). It is evident therefore that LRRK2 is associated with both PD and IBD, and increased LRRK2 activity may increase susceptibility to inflammation of the gastrointestinal tract which may play a role in PD.

Although there is evidence for a link between PD and IBD with LRRK2 at the interface, the concept of gut inflammation in PD has only been tested in LRRK2 animal models in two studies to date. The use of dextran sodium sulfate salt (DSS), a chemical colitogen with anticoagulant properties, is the most widely used method to model colitis in mice. A recent study demonstrated that the overexpression of *LRRK2* leads to increased susceptibility to DSS-induced colitis in mice (Takagawa et al., 2018). Furthermore, the normalization of LRRK2 kinase activity blocked the release of TNF by cultured cells from patients with CD with no *LRRK2*-mutations, suggesting that targeting LRRK2 activity could be a therapeutic approach for IBD regardless of whether a *LRRK2* risk allele is involved. However, it has previously been demonstrated that the down-regulation of *Lrrk2* enhances the susceptibility of mice to DSS-induced colitis (Liu et al., 2011), suggesting that a loss of LRRK2 is also sufficient to increase inflammation in the gut. Differences in experimental paradigm between these two studies make it difficult to conclude the role of LRRK2 activity in colitis. A large number of factors affect susceptibility to DSS and can modify results, making it difficult to compare across study designs. Factors such as the background strain of the experimental animal, age, microbial state (specific pathogen free vs. open cages), and the DSS treatment dosage and duration (Perse and Cerar, 2012) all influence outcomes of DSS-induced colitis studies. Furthermore, differences in the control mice used in the two studies may also contribute to these discrepancies. For example, it was observed by Takagawa and colleagues that littermate control mice manifested a different degree of colitis than mice directly obtained from the mouse supplier. Therefore assessment of the severity of colitis required utilization of control mice with identical genetic and microbiological features to the experimental mice. Future research utilizing consistent experimental colitis paradigms in LRRK2-animal models of PD are required in order to examine the effects of intestinal inflammation on PD-associated pathology in both the nigrostriatal pathway and gastrointestinal system.

One proposed model of the link between gastrointestinal inflammation and PD proposes that increased intestinal inflammation increases expression and aggregation of α-synuclein, which spreads to the brain via the vagal preganglionic innervation of the gut (Houser and Tansey, 2017; Rolli-Derkinderen et al., 2019). Such a model would be in accordance with the Braak staging system that suggests α-synuclein pathology initiates in the enteric neurons of the upper gastrointestinal tract and propagates to the CNS via the vagus nerve. There it progresses in a predictable fashion along a caudo-rostral axis in the brain (Braak et al., 2003a, b). Based on this model, to explore the role of LRRK2 and gut inflammation further, future research should investigate neurodegeneration and α-synuclein pathology in the ENS and the CNS of *LRRK2* transgenic mice subjected to DSS-induced colitis. Despite studies showing increased α-synuclein observed in the colon of CD patients, pathological changes such as aggregation are yet to be observed (Prigent et al., 2019). It is therefore of interest to investigate LRRK2 expression and phosphorylation in gastrointestinal samples of PD patients to determine the involvement of enteric α-synuclein in PD associated gastrointestinal inflammation.

### LRRK2, Systemic Inflammation and the Blood–Brain Barrier

Interestingly, it has recently been hypothesized that increased intestinal permeability and subsequent systemic inflammation may lead to the disruption of the BBB and, potentially, neuroinflammation and disruption of dopamine pathways (Houser and Tansey, 2017; Rolli-Derkinderen et al., 2019). Likewise, it has also been proposed that prolonged systemic inflammation caused by pathogen exposure and chronic immune cell activation in the periphery may amplify microglia activation, known as microglia priming (Perry and Teeling, 2013; Lee et al., 2017). Given the evidence for the role of LRRK2 in gastrointestinal inflammation, infection, peripheral immune responses and PD, LRRK2 may be situated in the center of this model (Figure 1).

A recently published study demonstrated that, when *R1441C* and *G2019S-LRRK2* mice are subjected to an acute, high-dose of LPS in the periphery, significant neuronal loss and an exacerbated immune response is observed in the brain and periphery relative to WT mice (Kozina et al., 2018). Furthermore, no infiltrating peripheral immune cells were observed in the parenchyma upon LPS stimulation and neuroinflammation was not directly mediated through resident microglia. It was therefore proposed that LPS-induced neuronal loss in *LRRK2* mutants are most likely initiated through circulating inflammatory mediators. It has also been observed that, whilst *G2019S-LRRK2* mice exhibit increased neuroinflammation upon LPS treatment, dopamine neuronal integrity was unaltered, implying that repeated exposure to inflammatory triggers may be needed in order for *LRRK2* mutations to cause dopaminergic neuronal loss (Schildt et al., 2019). Such data supports a role of LRRK2 in peripheral-to-centrally mediated immune signaling. Although the mechanisms connecting the peripheral immune response and neuroinflammation are not fully understood, increased circulating pro-inflammatory cytokines may induce a disruption of the BBB and passively diffuse and promote microglia-mediated inflammation and toxicity as a secondary effect (Alvarez-Arellano and Maldonado-Bernal, 2014; Bodea et al., 2014). Alternatively, cytokines may actively be transported via saturable transport systems on endothelial cells (Pan et al., 2011). It would be of interest to future research to determine if disruptions in BBB permeability induced by LRRK2-mediated peripheral inflammation is also accompanied by increased uptake of α-synuclein from circulation into the CNS, as has been observed in other models (Sui et al., 2014).

### LRRK2 Interacting Partners and Inflammation

Although a number of proteins have been reported to be directly regulated by LRRK2, few have been validated and replicated by numerous groups (Price et al., 2018). Recent studies have identified a subset of Rab GTPases as *bona fide* substrates of LRRK2 in cells (Steger et al., 2016, 2017; Fujimoto et al., 2018; Liu et al., 2018). The role of these Rab GTPases and LRRK2 in immune cells has been reviewed recently (Wallings and Tansey, 2019). However, of note here is the role of Rab GTPases in the regulation of transcytosis (Preston et al., 2014). Transcytosis is a type of transcellular transport in which macromolecules are transported across the interior of a cell. Maintaining a low rate of transcytosis in the endothelial cells that constitute the BBB is critical to maintaining a functioning barrier (Ayloo and Gu, 2019). Rab35, a known LRRK2 substrate, is instrumental in the docking and recycling of vesicles as well as transcytosis (Mrozowska and Fukuda, 2016). Interestingly, it has recently been demonstrated that LRRK2 mediates α-synuclein propagation via increased phosphorylation and activation of Rab35 (Bae et al., 2018). It is interesting to note that Rab35 expression is elevated in the serum of PD patients and in brain tissue of PD mouse models, including *G2019S-LRRK2* mice (Chiu et al., 2016). It is therefore feasible to hypothesize that increased LRRK2 activity may increase Rab35 activity in endothelial cells and subsequently elevate the rate of transcytosis, leading to the BBB becoming compromised, and is of interest to future research.

It is important to note that LRRK2 expression has been found to be expressed highly in human neutrophils (Fan et al., 2018). Neutrophils are first responders to sites of infection, where they utilize novel bacterial sensing pathways leading to phagocytosis and production of bactericidal factors (Witter et al., 2016). The LRRK2 kinase substrate, Rab10, has also been shown to be highly expressed and phosphorylated by LRRK2 in isolated human neutrophils (Fan et al., 2018). Rab10 is known to regulate phagosomal recycling (Chua and Tang, 2018) and up-regulates lysosomal secretion during lysosomal stress alongside Rab8, Rab7L1 and LRRK2 (Eguchi et al., 2018). Given that the intracellular killing of microorganisms in phagocytes such as neutrophils involves the fusion of lysosomes containing bactericidal factors with phagosomes, it would be of interest to future research to investigate LRRK2-regulation of immune responses in neutrophils and the effects of mutations in lysosome stress, pathogen control, and neuroinflammation.

LRRK2 and α-synuclein have been shown to share a complex relationship, and it seems that LRRK2 dysfunction can modulate α-synuclein and its relevant cellular pathways [reviewed in detail in Cresto et al. (2018)]. Furthermore, α-synuclein is implicated in neuroinflammation observed in PD, suggesting these two PD-related proteins may be associated in the context of inflammation. For example, activated microglia are observed in the midbrain of animals after intra-striatal injection of α−synuclein PFFs prior to dopaminergic neuronal loss (Duffy et al., 2018). Furthermore, the use of agonists of the Glucagon−like peptide−1 receptor (GLP1R), which inhibits microglia−induced activation of astrocytes, protects against α-synuclein toxicity in α-synuclein PFF mouse model of PD as well as the human *A53T* α-synuclein transgenic mouse model (Yun et al., 2018). As previously mentioned, *Lrrk2*-KO alters inflammatory gene changes in response to α−synuclein PFFs, suggesting LRRK2 is involved in the cellular pathways implicated in α−synuclein inflammation (Russo et al., 2019). This is supported by observed LRRK2 immunoreactivity in CD68^+^ cells in the SNpc which are recruited in response to α-synuclein transduction, with *Lrrk2*-KO decreasing this recruitment as well as microglia activation and dopaminergic neuronal loss (Daher et al., 2014). Furthermore, LRRK2 kinase inhibition attenuates neuroinflammation in *G2019S-LRRK2* transgenic rats after α−synuclein transduction (Daher et al., 2015). Whether such interactions between LRRK2 and α−synuclein is also seen in peripheral immune cells remains to be determined. Collectively such data, taken with the fact that LRRK2 is capable of modulating the propagation of α-synuclein (Bae et al., 2018), suggest LRRK2 dysfunction may influence α−synuclein and its pathology through mechanisms altering cellular functions and signaling pathways in the immune system.

## Conclusion

PD is typically thought of as a disease of the CNS. However, evidence discussed in this review emphasizes a crucial role of the immune system, both peripherally and centrally, in PD pathophysiology. It appears that LRRK2 plays a fundamental role in the regulation of inflammation in both the central and peripheral immune system, and therefore may be instrumental in PD-associated inflammation. Furthermore, LRRK2 may lie center stage in the cross-talk between the peripheral and central immune system, with increased inflammation in the gut or in response to pathogens with *LRRK2* mutations potentially leading to increased gut dysbiosis, BBB permeability and microglia priming.

One model of PD touched upon in this review was the “multiple-hit” hypothesis which identifies factors such as bacterial and viral infection and microbiome perturbations as triggering events of PD, with genetics and aging facilitating and exacerbating disease onset and progression (Westerlund et al., 2008; Gao et al., 2011). Given the evidence discussed implicating LRRK2 in the regulation of immune responses to pathogens, it is curious to speculate if epidemiological research would demonstrate increased rates of previous infections in *LRRK2*-PD patients relative to non-manifesting *LRRK2* carriers.

The LRRK2 kinase domain has become an appealing target for therapeutics, with increased LRRK2 kinase activity seen in PD mutations, and increased LRRK2 expression and kinase activity observed in sporadic patients (Di Maio et al., 2018; Atashrazm et al., 2019). The evidence discussed in this review suggests LRRK2-mediated inflammation may be an early event in PD and may therefore be a preventative target for the disease. However, it is important consider that a complete abolition of LRRK2 kinase activity in the peripheral immune system may have deleterious effects, with increased risk of infection and decreased pathogen control, as suggested by data from *Lrrk2*-KO models (Gardet et al., 2010; Zhang Q. et al., 2015; Liu et al., 2017; Wallings and Tansey, 2019). Therefore, such malignant side-effects would need to be taken into consideration if such inhibitors were to be therapeutically beneficial.

## Author Contributions

All authors listed have made a substantial, direct and intellectual contribution to the work, and approved it for publication.

## Conflict of Interest

The authors declare that the research was conducted in the absence of any commercial or financial relationships that could be construed as a potential conflict of interest.
